# Gradient Crystal Plasticity: A Grain Boundary Model for Slip Transmission

**DOI:** 10.3390/ma12223761

**Published:** 2019-11-15

**Authors:** Xiang-Long Peng, Gan-Yun Huang, Swantje Bargmann

**Affiliations:** 1 Department of Mechanics, School of Mechanical Engineering, Tianjin University, Tianjin 300350, China; 2 Chair of Solid Mechanics, School of Mechanical Engineering and Safety Engineering, University of Wuppertal, 42119 Wuppertal, Germany; bargmann@uni-wuppertal.de

**Keywords:** slip transmission, strain gradient, crystal plasticity, size effects, grain boundary

## Abstract

Interaction between dislocations and grain boundaries (GBs) in the forms of dislocation absorption, emission, and slip transmission at GBs significantly affects size-dependent plasticity in fine-grained polycrystals. Thus, it is vital to consider those GB mechanisms in continuum plasticity theories. In the present paper, a new GB model is proposed by considering slip transmission at GBs within the framework of gradient polycrystal plasticity. The GB model consists of the GB kinematic relations and governing equations for slip transmission, by which the influence of geometric factors including the misorientation between the incoming and outgoing slip systems and GB orientation, GB defects, and stress state at GBs are captured. The model is numerically implemented to study a benchmark problem of a bicrystal thin film under plane constrained shear. It is found that GB parameters, grain size, grain misorientation, and GB orientation significantly affect slip transmission and plastic behaviors in fine-grained polycrystals. Model prediction qualitatively agrees with experimental observations and results of discrete dislocation dynamics simulations.

## 1. Introduction

Fine-grained polycrystalline materials with grain sizes ranging from hundreds of nanometers to tens of microns can be widely used as structural or functional materials in small-scaled engineering such as microdevices and microelectromechanical systems. Both their precision manufacturing and processing and their safety and reliability in practical applications entail a deep understanding of the plastic behavior in fine-grained polycrystals. As found in various experiments [1,2,3,4,5,6], if the average grain size decreases to the micrometer range or even smaller, the plastic behavior in crystals such as macroscopic yield strength and strain hardening rate become strongly size-dependent. Microscopically, plastic deformation in crystals mainly originates from the collective behavior of a vast number of dislocations, implying that size-dependent plasticity is closely related to the distinctive dislocation activities in small-scaled crystals. Since the grain boundaries (GBs) to volume ratio in fine-grained polycrystals is extremely high, interaction between dislocations and GBs may be an important, and in some situations, dominant deformation mechanism therein. From numerous in situ experiments as reviewed in [7] and atomistic simulations as reviewed in [8], dislocations interact with GBs in several different ways. Due to the crystallographic incompatibility between adjacent grains, GBs act as obstacles, impeding dislocations nucleated within the grain interior, which results in dislocation pile-ups at GBs [9]. In response to stress concentrations resulting from dislocation pile-ups, GBs may act as dislocation sinks or sources, absorbing the leading dislocation in the pile-up or emitting new dislocations onto the slip planes in the adjacent grains [9,10,11]. Under certain conditions, dislocations are transferred through GBs in a direct or indirect manner with the generation of residual GB defects for the conservation of Burgers vectors, which is also known as slip transmission [9,12,13,14]. Various factors such as the initial GB structure, the GB orientation, the grain misorientation, and the stress state near the GB affect dislocation-GB interactions [7,15]. As illustrated by experiments [16,17] and discrete dislocation dynamics (DDD) simulations [18,19,20], dislocation-GB interactions can significantly affect macroscopic plastic behaviors in fine-grained polycrystals. Moreover, due to the GB constraint, plastic deformation in fine-grained polycrystals is strongly heterogeneous, which results in the accumulation of geometrically necessary dislocations (GNDs) stored to accommodate lattice curvature [21]. There exists a close connection between GNDs and size-dependent plasticity in crystals [22]. Conventional plasticity theories fail to capture dislocation-GB interactions and the influence of GNDs and are incapable of predicting size-dependent plasticity in fine-grained polycrystals. Thus, it is vital to properly model the above microscopic dislocation-relevant mechanisms, especially dislocation-GB interaction mechanisms at the continuum level, so as to construct non-classical plasticity theories aimed at the accurate characterization of size-dependent plasticity in fine-grained polycrystals.

In the last three decades or so, many efforts have been devoted to develop various strain gradient plasticity theories with consideration of the influence of plastic strain gradient or GNDs, which are capable of predicting some experimentally observed size effects [23,24,25,26,27]. In higher-order strain gradient plasticity theories [28,29,30,31,32,33,34,35], in addition to the traditional force balance equations, microscopic governing equations involving higher-order stresses/microstresses or back stresses resulting from interactions between GNDs are introduced. Consequently, additional microscopic boundary conditions at GBs/surfaces are required for the completeness of the mathematical framework. This provides the possibility to incorporate the GB/surface-dislocation interactions into those continuum theories. Within the framework of high-order strain gradient plasticity theories, some GB/surface models have been proposed. Two frequently used idealized GB/surface conditions are the microhard or microclamped model (corresponding to impenetrable GBs/surfaces) and the microfree model (corresponding to freely penetrable GB/surfaces). Additionally, some attention has been paid to develop intermediate GB/surface models for realistic GBs/surfaces with finite resistance against dislocation gliding. From the viewpoint of thermodynamics, the GB/surface-dislocation interaction is of both energetic and dissipative nature since emission, absorption, and transmission of dislocations may lead to the storage of residual defects/surface steps at GBs/surfaces, and such processes are resistive. Thus, the existing intermediate GB/surface models can be classified into those of dissipative ones [36,37,38,39], energetic ones [40,41,42,43,44,45], and both energetic and dissipative ones [25,46,47,48,49,50,51,52]. The majority of these existing surface/GB models are phenomenological. A few physically based GB/surface models [46,47,48,52] capture GB/surface effects to some extent.

Due the complication of interaction between dislocations and GBs, further modeling efforts are still needed. In fact, different forms of dislocation-GB interaction such as GB absorbing, emitting, and transmitting dislocations may affect the macroscopic plastic behaviors in fine-grained polycrystals in different ways [53]. Therefore, it is necessary to distinguish these different GB mechanisms in GB models.

Based on the above background, the aim of the present work is to model slip transmission at the GB by considering the underlying physical mechanism. Within the framework of finite deformation gradient polycrystal plasticity, a new GB model for slip transmission is proposed. The GB model consisting of the GB kinematic relations and microscopic force balance equations for slip transmission captures the influence of geometric factors including the misorientation between the incoming and outgoing slip systems and GB orientation, GB defects, and stress state at GBs. The model is applied to study the plastic behavior of a bicrystal thin film under plane constrained shear. Results predicted by the model qualitatively agrees with those from experimental observations and DDD simulations.

## 2. Model Framework

### 2.1. Bulk Kinematics: Finite Deformation Gradient Polycrystal Plasticity

Following [54], in finite deformation plasticity, the deformation gradient tensor F is assumed to be multiplicatively decomposed into an elastic part $F^{e}$ and a plastic part $F^{p}$,

(1) $$F=F^{e}\cdot F^{p}.$$

Consider that plastic deformation induces no change to volume, one obtains det$F^{p}=1$ with det being the determinant operator. In the following, if necessary, quantities in the intermediate and those in the current configuration will be identified by the subscripts i and c, respectively.

The velocity gradient tensor L is defined as

(2) $$L=\nabla_{c}\dot{u}=\dot{F}\cdot F^{-1}=L^{e}+F^{e}\cdot L^{p}\cdot F^{e-1},$$

where ${\left[\nabla a\right]}_{ij}=a_{i,j}$ represents the gradient of a vector a, u is the displacement vector, the superposed dot denotes time derivative, and the superscript −1 denotes the inverse of a tensor. The elastic velocity gradient $L^{e}$ and the plastic velocity gradient $L^{p}$ are expressed as

(3) $$L^{e}={\dot{F}}^{e}\cdot F^{e-1},$$

(4) $$L^{p}={\dot{F}}^{p}\cdot F^{p-1}.$$

Studying polycrystals, in each grain, a set of slip systems is introduced. For each slip system α, a unit vector of slip direction $s^{\left(\alpha \right)}$ and a unit vector of slip plane normal $m^{\left(\alpha \right)}$ attached to the lattice space in the intermediate configuration are defined. $s^{\left(\alpha \right)}$ and $m^{\left(\alpha \right)}$ are assumed to be unaltered from the reference configuration to the intermediate configuration. Following [55], the plastic velocity gradient consisting of contributions from all active slip systems reads

(5) $$L^{p}=\sum_{\alpha }{\dot{\gamma }}^{\left(\alpha \right)}s^{\left(\alpha \right)}⊗m^{\left(\alpha \right)},$$

with ${\dot{\gamma }}^{\left(\alpha \right)}$ being the plastic slip rate of the slip system α.

The involved strain measures are the Green-Lagrange strain tensor E and the right Cauchy-Green stretch tensor C, and their elastic parts $E^{e}$ and $C^{e}$, which are expressed as

(6) $$E=\frac{1}{2}\left[C-I\right],\;C=F^{T}\cdot F,$$

(7) $$E^{e}=\frac{1}{2}\left[C^{e}-I\right],\;C^{e}=F^{\mathrm{eT}}\cdot F^{e}.$$

The superscript T denotes the transpose of a tensor, and I is the second order identity tensor.

For the slip system α, the evolutions of the edge and screw GND densities (The GND density characterizes the net density of polarized dislocations. Once the slip rate gradient is given, the edge and screw GND densities can be evaluated by Equations (8) and (9), respectively). $\rho_{i}^{\mathrm{ge}(\alpha )}$ and $\rho_{i}^{\mathrm{gs}(\alpha )}$ defined in the intermediate configuration are expressed as

(8) $${\dot{\rho }}_{i}^{\mathrm{ge}(\alpha )}=-\frac{1}{b^{\left(\alpha \right)}}\nabla_{i}{\dot{\gamma }}^{\left(\alpha \right)}\cdot s^{\left(\alpha \right)},$$

(9) $${\dot{\rho }}_{i}^{\mathrm{gs}(\alpha )}=\frac{1}{b^{\left(\alpha \right)}}\nabla_{i}{\dot{\gamma }}^{\left(\alpha \right)}\cdot p^{\left(\alpha \right)},$$

where $\nabla_{i}\left(\cdot \right)=F^{p-T}\cdot \nabla_{r}\left(\cdot \right)$, $b^{\left(\alpha \right)}$ is the magnitude of Burgers vector, and $p^{\left(\alpha \right)}=s^{\left(\alpha \right)}\times m^{\left(\alpha \right)}$. In addition, the initial conditions are $\rho_{i}^{\mathrm{ge}(\alpha )}{|}_{t=0}=\rho_{0i}^{\mathrm{ge}(\alpha )}$ and $\rho_{i}^{\mathrm{gs}(\alpha )}{|}_{t=0}=\rho_{0i}^{\mathrm{gs}(\alpha )}$, with $\rho_{0i}^{\mathrm{ge}(\alpha )}$ and $\rho_{0i}^{\mathrm{gs}(\alpha )}$ being the initial GND densities.

### 2.2. Kinematic Relations at GBs: Slip Transmission

**Figure 1:**
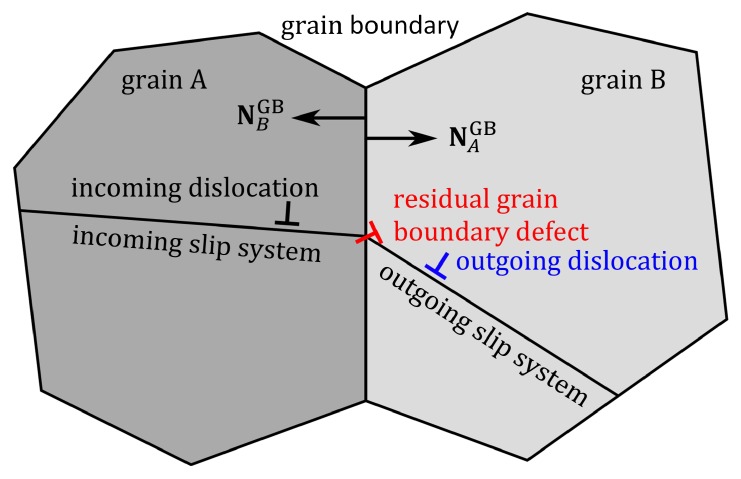
Schematic of slip transmission: dislocations from an incoming slip system in grain *A* propagate through the grain boundary $\Omega_{AB}$ onto the outgoing slip system in grain *B*. The transmission of each dislocation produces a residual grain boundary defect.

As illustrated in Figure 1, slip transmission occurs in a way that dislocations on the incoming slip systems in grain *A* are transferred through the GB $\Omega_{AB}$ onto the corresponding outgoing slip systems in grain *B*, during which the transmission of each dislocation produces a residual GB defect for the conservation of Burgers vector. In the following, subscripts *A* and *B* are used to identify quantities in grains *A* and *B* respectively. The outward normal unit vectors at the GB of grains *A* and *B* are denoted as $N_{A}^{\mathrm{GB}}$ and $N_{B}^{\mathrm{GB}}$ in the intermediate configuration. In continuum crystal plasticity, it is assumed that slip planes and dislocations are continuously distributed. Thus, we consider the slip transmission as a point-level process at GBs. Generally, a specified incoming slip system may correspond to several different outgoing slip systems, and vice versa. Thus, for an incoming slip system α, the total number of incoming dislocations per unit time $n_{A}^{\left(\alpha \right)}$ transmitted through a point $x_{\mathrm{GB}}$ on the GB $\Omega_{AB}$ is expressed as

(10) $$n_{A}^{\left(\alpha \right)}=\sum_{\beta }n_{AB}^{\mathrm{in}(\alpha \beta )},$$

where $n_{AB}^{\mathrm{in}(\alpha \beta )}$ denotes the number of incoming dislocations per unit time during the slip transmission process between the incoming slip system α and the outgoing slip system β. Accordingly, for an outgoing slip system β the total number of outgoing dislocations per unit time $n_{B}^{\left(\beta \right)}$ transmitted from the point $x_{\mathrm{GB}}$ on the GB $\Omega_{AB}$ is expressed as

(11) $$n_{B}^{\left(\beta \right)}=\sum_{\alpha }n_{AB}^{\mathrm{out}(\alpha \beta )},$$

where $n_{AB}^{\mathrm{out}(\alpha \beta )}$ is the number of outgoing dislocations per unit time during the slip transmission process between the incoming slip system α and the outgoing slip system β. As illustrated in Figure 1, for a specified slip transmission process between the incoming slip system α and the outgoing slip system β, each incoming dislocation may result in one outgoing dislocation. Therefore, the number of incoming dislocations per unit time equals the number of the outgoing dislocations per unit time, i.e.,

(12) $$n_{AB}^{\mathrm{in}(\alpha \beta )}=n_{AB}^{\mathrm{out}(\alpha \beta )}.$$

Given the number of dislocations per unit time $n^{\left(\alpha \right)}$ gliding through a point x and the average distance between slip planes $L^{\left(\alpha \right)}$, the plastic slip rate ${\dot{\gamma }}^{\left(\alpha \right)}$ for a slip system α at that point can be expressed as

(13) $${\dot{\gamma }}^{\left(\alpha \right)}=\frac{n^{\left(\alpha \right)}b^{\left(\alpha \right)}}{L^{\left(\alpha \right)}}.$$

From Equations (10)–(13), the plastic slip rates ${\dot{\gamma }}_{A}^{\left(\alpha \right)}$ and ${\dot{\gamma }}_{B}^{\left(\beta \right)}$ for the incoming slip system α and the outgoing slip system β at the GB point $x_{\mathrm{GB}}$ are written as

(14) $${\dot{\gamma }}_{A}^{\left(\alpha \right)}=\sum_{\beta }{\dot{\gamma }}_{AB}^{\left(\alpha \beta \right)},\;{\dot{\gamma }}_{B}^{\left(\beta \right)}=\sum_{\alpha }D_{AB}^{\left(\alpha \beta \right)}{\dot{\gamma }}_{AB}^{\left(\alpha \beta \right)},$$

with

(15) $${\dot{\gamma }}_{AB}^{\left(\alpha \beta \right)}=\frac{n_{AB}^{\mathrm{in}(\alpha \beta )}b_{A}^{\left(\alpha \right)}}{L_{A}^{\left(\alpha \right)}},\;D_{AB}^{\left(\alpha \beta \right)}=\frac{b_{B}^{\left(\beta \right)}L_{A}^{\left(\alpha \right)}}{b_{A}^{\left(\alpha \right)}L_{B}^{\left(\beta \right)}}.$$

${\dot{\gamma }}_{AB}^{\left(\alpha \beta \right)}$ and $D_{AB}^{\left(\alpha \beta \right)}\gamma_{AB}^{\left(\alpha \beta \right)}$ are the components of ${\dot{\gamma }}_{A}^{\left(\alpha \right)}$ and ${\dot{\gamma }}_{B}^{\left(\beta \right)}$ contributed by the slip transmission process between the incoming slip system α and the outgoing slip system β. Equation (14) represents the GB kinematic relation in the present model.

By taking advantage of the balance of Burgers vector at the GB [46] and the GB kinematic relation in Equation (14), the rate of the density of residual GB defects $G_{ABi}^{\left(\alpha \beta \right)}$ contributed by the slip transmission between the incoming slip system α and the outgoing slip system β in the intermediate configuration can be expressed as

(16) $${\dot{G}}_{ABi}^{\left(\alpha \beta \right)}=-{\dot{\gamma }}_{AB}^{\left(\alpha \beta \right)}M_{ABi}^{\left(\alpha \beta \right)}$$

with

(17) $$M_{ABi}^{\left(\alpha \beta \right)}=\left[s_{A}^{\left(\alpha \right)}⊗\left[m_{A}^{\left(\alpha \right)}\times N_{Ai}^{\mathrm{GB}}\right]+D_{AB}^{\left(\alpha \beta \right)}s_{B}^{\left(\beta \right)}⊗\left[m_{B}^{\left(\beta \right)}\times N_{Bi}^{\mathrm{GB}}\right]\right].$$

### 2.3. Balance Equations and Boundary Conditions

In the present work, the work-conjugate gradient crystal plasticity framework is adopted, and, hence, the balance equations and boundary conditions are derived via the principle of virtual power. Without loss of generality, a bicrystal domain consisting of grains *A* and *B* separated by the GB $\Omega_{AB}$ is considered. The total volume and the total surface area of the domain are $V_{i}=V_{Ai}⋃V_{Bi}$ and $S_{i}=S_{Ai}⋃S_{Bi}$. Following [31], in the intermediate configuration, the second Piola-Kirchhof stress tensor $S^{e}$ power-conjugate to the rate of the Green-Lagrange strain tensor ${\dot{E}}^{e}$, the microforce $\pi_{i}^{\left(\alpha \right)}$ power-conjugate to the slip rate ${\dot{\gamma }}^{\left(\alpha \right)}$ and the microstress $\xi_{i}^{\left(\alpha \right)}$ power-conjugate to the slip rate gradient $\nabla_{i}{\dot{\gamma }}^{\left(\alpha \right)}$ for each slip system α contribute to the internal power in the grain interiors. Following [52], the contribution to the internal power by the surface microforce $\eta_{i}^{S(\alpha )}$ power-conjugate to the slip rate $\dot{\gamma }$ at the surface is considered to account for the influence of dislocation absorption by surfaces. In addition, to incorporate the influence of slip transmission between the incoming slip system α and the outgoing slip system β into the present theory, the contribution by the GB microforce $\eta_{ABi}^{\mathrm{GB}(\alpha \beta )}$ power-conjugate to ${\dot{\gamma }}_{AB}^{\left(\alpha \beta \right)}$ at the GB is introduced. It is assumed that no GB sliding or opening occurs. In other words, displacements are continuous at the GB. Therefore, there is no macroscopic power contribution at the GB. Thus, the total internal power $P^{\mathrm{int}}$ in the intermediate configuration is

(18) $$\begin{array}{cc} P^{\mathrm{int}} & =\int_{V_{i}}S^{e}:{\dot{E}}^{e}dV_{i}+\sum_{\alpha }\int_{V_{i}}\left[\pi_{i}^{\left(\alpha \right)}{\dot{\gamma }}^{\left(\alpha \right)}+\xi_{i}^{\left(\alpha \right)}\cdot \nabla_{i}{\dot{\gamma }}^{\left(\alpha \right)}\right]dV_{i}\\ \; & +\sum_{\alpha ,\beta }\int_{\Omega_{ABi}}\eta_{ABi}^{\mathrm{GB}(\alpha \beta )}{\dot{\gamma }}_{AB}^{\left(\alpha \beta \right)}d\Omega_{ABi}+\sum_{\alpha }\int_{S_{i}}\eta_{i}^{S(\alpha )}{\dot{\gamma }}^{\left(\alpha \right)}dS_{i}, \end{array}$$

where the second Piola-Kirchholf stress tensor $S^{e}$ is defined as

(19) $$S^{e}=F^{e-1}\cdot J\sigma \cdot F^{e-T}$$

with σ being the Cauchy stress tensor, and $J=\det F^{e}$. In the absence of body forces, the traditional traction $T_{i}$ power-conjugate to the velocity $\dot{u}$ at the surface and the microtraction $\Xi_{i}^{\left(\alpha \right)}$ power-conjugate to the plastic slip rate ${\dot{\gamma }}^{\left(\alpha \right)}$ at the surface contribute to the external power $P^{\mathrm{ext}}$, which gives

(20) $$P^{\mathrm{ext}}=\int_{S_{i}}T_{i}\cdot \dot{u}dS_{i}+\sum_{\alpha }\int_{S_{i}}\Xi_{i}^{\left(\alpha \right)}{\dot{\gamma }}^{\left(\alpha \right)}dS_{i}.$$

According to the principle of virtual power, the variation of the internal power with respect to the velocity $\dot{u}$ and the plastic slip rate ${\dot{\gamma }}^{\left(\alpha \right)}$ equals that of the external power, resulting in

(21) $$\begin{array}{cc} \; & \int_{V_{i}}S^{e}:\delta {\dot{E}}^{e}dV_{i}+\sum_{\alpha }\int_{V_{i}}\left[\pi_{i}^{\left(\alpha \right)}\delta {\dot{\gamma }}^{\left(\alpha \right)}+\xi_{i}^{\left(\alpha \right)}\cdot \nabla_{i}\delta {\dot{\gamma }}^{\left(\alpha \right)}\right]dV_{i}\\ \; & +\sum_{\alpha ,\beta }\int_{\Omega_{ABi}}\eta_{ABi}^{\mathrm{GB}(\alpha \beta )}\delta {\dot{\gamma }}_{AB}^{\left(\alpha \beta \right)}d\Omega_{ABi}+\sum_{\alpha }\int_{S_{i}}\eta_{i}^{S(\alpha )}\delta {\dot{\gamma }}^{\left(\alpha \right)}dS_{i}\\ \; & =\int_{S_{i}}T_{i}\cdot \delta \dot{u}dS_{i}+\sum_{\alpha }\int_{S_{i}}\Xi_{i}^{\left(\alpha \right)}\delta {\dot{\gamma }}^{\left(\alpha \right)}dS_{i}. \end{array}$$

Based on Equations (2), (3), (5) and (7), the variation of ${\dot{E}}^{e}$ is expressed as

(22) $$\begin{array}{cc} \delta {\dot{E}}^{e} & =\frac{1}{2}\left[F^{\mathrm{eT}}\cdot \nabla_{i}\delta \dot{u}-\sum_{\alpha }C^{e}\cdot s^{\left(\alpha \right)}⊗m^{\left(\alpha \right)}\delta {\dot{\gamma }}^{\left(\alpha \right)}\right]\\ \; & +\frac{1}{2}{\left[F^{\mathrm{eT}}\cdot \nabla_{i}\delta \dot{u}-\sum_{\alpha }C^{e}\cdot s^{\left(\alpha \right)}⊗m^{\left(\alpha \right)}\delta {\dot{\gamma }}^{\left(\alpha \right)}\right]}^{T}. \end{array}$$

Substituting Equation (22) into Equation (21) and using the GB kinematic relation (14) and the divergence theorem, one can rewrite Equation (21) as

(23) $$\begin{array}{cc} \; & \int_{V_{i}}{\mathrm{Div}}_{i}\left(F^{e}\cdot S^{e}\right)\cdot \delta \dot{u}dV_{i}-\sum_{\alpha }\int_{V_{i}}\left[{\mathrm{Div}}_{i}\xi_{i}^{\left(\alpha \right)}+s^{\left(\alpha \right)}\cdot M\cdot m^{\left(\alpha \right)}-\pi_{i}^{\left(\alpha \right)}\right]\delta {\dot{\gamma }}^{\left(\alpha \right)}dV_{i}\\ \; & +\sum_{\alpha ,\beta }\int_{\Omega_{ABi}}\left[\xi_{Ai}^{\left(\alpha \right)}\cdot N_{Ai}^{\mathrm{GB}}+D_{AB}^{\left(\alpha \beta \right)}\xi_{Bi}^{\left(\beta \right)}\cdot N_{Bi}^{\mathrm{GB}}+\eta_{ABi}^{\mathrm{GB}(\alpha \beta )}\right]\delta {\dot{\gamma }}_{ABi}^{\left(\alpha \beta \right)}d\Omega_{ABi}\\ \; & +\sum_{\alpha }\int_{S_{i}}\left[\xi_{i}^{\left(\alpha \right)}\cdot N_{i}^{S}+\eta_{i}^{S(\alpha )}-\Xi_{i}^{\left(\alpha \right)}\right]\delta {\dot{\gamma }}^{\left(\alpha \right)}dS_{i}\\ \; & +\int_{S_{i}}\left[\left[F^{e}\cdot S^{e}\right]\cdot N_{i}^{S}-T_{i}\right]\cdot \delta \dot{u}dS_{i}=0, \end{array}$$

where $M=C^{e}\cdot S^{e}$ is the Mandel stress tensor, and $N_{i}^{S}$ is the surface outward normal unit vector. Considering that Equation (23) should be satisfied for arbitrary $\delta \dot{u}$, one obtains the following traditional balance of momentum and the corresponding standard traction condition in the intermediate configuration,

(24) $${\mathrm{Div}}_{i}\left(F^{e}\cdot S^{e}\right)=0$$

and

(25) $$\left[F^{e}\cdot S^{e}\right]\cdot N_{i}^{S}=T_{i}$$

at the part of surface $S_{i}^{T}$ with prescribed traction. Further, given the validity of Equation (23) for arbitrary $\delta {\dot{\gamma }}^{\left(\alpha \right)}$ and $\delta {\dot{\gamma }}_{ABi}^{\left(\alpha \beta \right)}$, microscopic balance equations in the bulk and the associated microscopic boundary conditions at the surface/GB are expressed as

(26) $${\mathrm{Div}}_{i}\xi_{i}^{\left(\alpha \right)}+\tau_{i}^{\left(\alpha \right)}-\pi_{i}^{\left(\alpha \right)}=0,$$

with $\tau_{i}^{\left(\alpha \right)}=s^{\left(\alpha \right)}\cdot M\cdot m^{\left(\alpha \right)}$ being the Schmid stress, and

(27) $$\xi_{i}^{\left(\alpha \right)}\cdot N_{i}^{S}+\eta_{i}^{S(\alpha )}=\Xi_{i}^{\left(\alpha \right)},$$

(28) $$\xi_{Ai}^{\left(\alpha \right)}\cdot N_{Ai}^{\mathrm{GB}}+D_{AB}^{\left(\alpha \beta \right)}\xi_{Bi}^{\left(\beta \right)}\cdot N_{Bi}^{\mathrm{GB}}+\eta_{ABi}^{\mathrm{GB}(\alpha \beta )}=0.$$

As indicated in [52], Equation (27) is regarded as the governing equation for dislocation absorption by surfaces. In the present GB model, Equation (28) acts as the governing equation for the slip transmission process between the incoming slip system α in grain *A* and the outgoing slip system β in grain *B* at the GB, in which the normal components of the microstresses $\xi_{Ai}^{\left(\alpha \right)}\cdot N_{Ai}^{\mathrm{GB}}$ and $\xi_{Bi}^{\left(\beta \right)}\cdot N_{Bi}^{\mathrm{GB}}$ from both grains are treated as the driving force for slip transmission.

In some existing GB models [46,47], each slip system possesses an independent microscopic force balance equation at the GB, and, hence, the correlation between plastic slips at one side of the GB and those at the other side is not directly reflected. However, during slip transmission, the plastic slip for the incoming slip system and that for the outgoing slip system should be coupled, which is captured in the present work. In the present GB model, an incoming slip system α in grain *A* and the corresponding outgoing slip system β in grain *B* involved in a specified slip transmission process share a GB governing equation, and the GB kinematic relation (14) serves as an additional boundary condition. In addition, since microstresses from both grains are involved in the GB governing Equation (28), the intergranular interaction between dislocations from the two adjacent grains at the GB is naturally considered.

### 2.4. Constitutive Relations

#### 2.4.1. Bulk Constitutive Relations: Gradient Crystal Plasticity

The elastic behavior of the crystal is captured by a compressible neo-Hookean material model, and, hence the hyperelastic constitutive relation for the second Piola-Kirchhof stress $S^{e}$ is given by

(29) $$S^{e}=\mu I+\left[\lambda \ln\left(J\right)-\mu \right]C^{e-1},$$

where $J=\det F^{e}=\sqrt{\det C^{e}}$, λ=2νμ/[1−2ν], μ is the shear modulus, and ν is Poisson’s ratio.

The microstress $\xi_{i}^{\left(\alpha \right)}$ generally consisting of an energetic part and a dissipative part [56,57,58] is assumed to be purely energetic for the sake of simplicity. The constitutive relation for $\xi_{i}^{\left(\alpha \right)}$ based on the elastic interaction between GNDs within the same slip system and those from different slip systems suggested in [59] is adopted here, i.e.,

(30) $$\xi_{i}^{\left(\alpha \right)}=\frac{\mu b^{\left(\alpha \right)}l^{2}}{8\left[1-\nu \right]}\sum_{\beta }\rho_{i}^{\mathrm{ge}(\beta )}B^{e(\alpha \beta )}+\frac{\mu b^{\left(\alpha \right)}l^{2}}{4}\sum_{\beta }\rho_{i}^{\mathrm{gs}(\beta )}B^{s(\alpha \beta )}$$

with

(31) $$\begin{array}{cc} B^{e(\alpha \beta )} & =\left[3\left[s^{\left(\alpha \right)}\cdot s^{\left(\beta \right)}\right]\left[m^{\left(\alpha \right)}\cdot s^{\left(\beta \right)}\right]+\left[s^{\left(\alpha \right)}\cdot m^{\left(\beta \right)}\right]\left[m^{\left(\alpha \right)}\cdot m^{\left(\beta \right)}\right]\right.\\ \; & \left.+4\nu \left[s^{\left(\alpha \right)}\cdot p^{\left(\beta \right)}\right]\left[m^{\left(\alpha \right)}\cdot p^{\left(\beta \right)}\right]\right]m^{\left(\beta \right)}\\ \; & -\left[\left[s^{\left(\alpha \right)}\cdot s^{\left(\beta \right)}\right]\left[m^{\left(\alpha \right)}\cdot m^{\left(\beta \right)}\right]+\left[s^{\left(\alpha \right)}\cdot m^{\left(\beta \right)}\right]\left[m^{\left(\alpha \right)}\cdot s^{\left(\beta \right)}\right]\right]s^{\left(\beta \right)},\\ B^{s(\alpha \beta )} & =-\left[\left[s^{\left(\alpha \right)}\cdot p^{\left(\beta \right)}\right]\left[m^{\left(\alpha \right)}\cdot s^{\left(\beta \right)}\right]+\left[s^{\left(\alpha \right)}\cdot s^{\left(\beta \right)}\right]\left[m^{\left(\alpha \right)}\cdot p^{\left(\beta \right)}\right]\right]m^{\left(\beta \right)}\\ \; & +\left[\left[s^{\left(\alpha \right)}\cdot s^{\left(\beta \right)}\right]\left[m^{\left(\alpha \right)}\cdot m^{\left(\beta \right)}\right]+\left[s^{\left(\alpha \right)}\cdot m^{\left(\beta \right)}\right]\left[m^{\left(\alpha \right)}\cdot s^{\left(\beta \right)}\right]\right]p^{\left(\beta \right)}, \end{array}$$

where *l* denotes the radius of the domain within which the interaction between GNDs is considered.

A visco-plastic power-law relation is adopted for the dissipative microforce $\pi_{i}^{\left(\alpha \right)}$, i.e.,

(32) $$\pi_{i}^{\left(\alpha \right)}=R_{i}^{b(\alpha )}{\left[\frac{{\dot{\gamma }}^{\left(\alpha \right)}}{{\dot{\gamma }}_{0}^{b}}\right]}^{m_{b}}\mathrm{sgn}\left({\dot{\gamma }}^{\left(\alpha \right)}\right),$$

where sgn() is the sign function, $m_{b}$ is the rate-sensitivity exponent in the bulk, and ${\dot{\gamma }}_{0}^{b}$ is the bulk reference slip rate. The slip resistance $R_{i}^{b(\alpha )}$ resulting from the interaction between statistically stored dislocations (SSDs) has the following linear hardening form,

(33) $$R_{i}^{b(\alpha )}=R_{0}^{b(\alpha )}+\sum_{\beta }H^{b(\alpha \beta )}\gamma_{\mathrm{acc}}^{\left(\beta \right)},$$

where $\gamma_{\mathrm{acc}}^{\left(\beta \right)}=\int \left|{\dot{\gamma }}^{\left(\beta \right)}\right|dt$ is the accumulated plastic slip, $R_{0}^{b(\alpha )}$ is the initial slip resistance, and $H^{b(\alpha \beta )}$ is the local hardening modulus. Similar to some existing works [52,58], in the present model, the evolution of the SSD density which quantifies the total non-polarized dislocation density is not explicitly considered. Instead, the influence of SSDs is reflected by the dependence of the slip resistance $R_{i}^{b(\alpha )}$ on the accumulated plastic slip which acts as a measure of the SSD density.

Inserting Equation (32) into Equation (26), one can rewrite the microscopic balance equation as

(34) $${\dot{\gamma }}^{\left(\alpha \right)}={\dot{\gamma }}_{0}^{b}{\left[\frac{\left|{\mathrm{Div}}_{i}\xi_{i}^{\left(\alpha \right)}+\tau_{i}^{\left(\alpha \right)}\right|}{R_{i}^{b(\alpha )}}\right]}^{\frac{1}{m_{b}}}\mathrm{sgn}\left({\mathrm{Div}}_{i}\xi_{i}^{\left(\alpha \right)}+\tau_{i}^{\left(\alpha \right)}\right),$$

which acts as the evolution equation for the plastic slip in the bulk.

#### 2.4.2. Surface Constitutive Relation: Dislocation Absorption by Surfaces

The surface model with consideration of the influence of dislocation absorption by surfaces in [52] is summarized here. In order to consider energetic and dissipative surface effects respectively, the surface microforce $\eta_{i}^{S(\alpha )}$ is divided into an energetic part $\eta_{i}^{\mathrm{Sen}(\alpha )}$ and a dissipative part $\eta_{i}^{\mathrm{Sdis}(\alpha )}$. The energetic surface microforce $\eta_{i}^{\mathrm{Sen}(\alpha )}$ is expressed as

(35) $$\begin{array}{cc} \eta_{i}^{\mathrm{Sen}(\alpha )} & =\Gamma_{1}^{\left(\alpha \right)}\left|s^{\left(\alpha \right)}\cdot N_{i}^{S}\right|\mathrm{sgn}\left(\gamma^{\left(\alpha \right)}\right)\\ \; & +\sum_{\beta }\Gamma_{2}^{\left(\alpha \beta \right)}\left|s^{\left(\alpha \right)}\cdot N_{i}^{S}\right|\left|m^{\left(\alpha \right)}\times N_{i}^{S}\right|\left|s^{\left(\beta \right)}\cdot N_{i}^{S}\right|\left|m^{\left(\beta \right)}\times N_{i}^{S}\right|\gamma^{\left(\beta \right)}, \end{array}$$

which is derived by considering the change of the surface energy due to the formation of surface steps after dislocation absorption by surfaces. In Equation (35), $\Gamma_{1}^{\left(\alpha \right)}$ denotes the surface free energy per unit surface step area, and $\Gamma_{2}^{\left(\alpha \beta \right)}$ is the energetic surface hardening modulus measuring the interaction strength between surface steps from slip systems α and β. The dissipative microforce $\eta_{i}^{\mathrm{Sdis}(\alpha )}$ accounting for the dissipative resistance against dislocation absorption by surfaces has a visco-plastic power-law form,

(36) $$\eta_{i}^{\mathrm{Sdis}(\alpha )}=R_{i}^{S(\alpha )}{\left[\frac{{\dot{\gamma }}^{\left(\alpha \right)}}{{\dot{\gamma }}_{0}^{S}}\right]}^{m_{S}}\mathrm{sgn}\left({\dot{\gamma }}^{\left(\alpha \right)}\right),$$

where ${\dot{\gamma }}_{0}^{S}$ is the reference slip rate at the surface, and $m_{S}$ is the surface rate-sensitivity exponent. The surface slip resistance $R_{i}^{S(\alpha )}$ is expressed as

(37) $$R_{i}^{S(\alpha )}=R_{0}^{S(\alpha )}+\sum_{\beta }H^{S(\alpha \beta )}\gamma_{\mathrm{acc}}^{S(\beta )},$$

where $\gamma_{\mathrm{acc}}^{S(\beta )}=\int \left|{\dot{\gamma }}^{\left(\beta \right)}\right|dt$ is the accumulated slip at the surface, $R_{0}^{S(\alpha )}$ is the initial surface slip resistance depending on the initial density of surface defects, and $H^{S(\alpha \beta )}$ is the dissipative surface hardening modulus. The surface parameters may depend on the initial surface state such as surface coatings, oxide layers or initial surface defects.

The higher-order traction $\Xi_{i}^{\left(\alpha \right)}$ is ignored in the following. Then, by taking advantage of Equation (36), the microscopic surface boundary condition Equation (27) is rewritten as

(38) $${\dot{\gamma }}^{\left(\alpha \right)}={\dot{\gamma }}_{0}^{S}{\left[\frac{\left|-\xi_{i}^{\left(\alpha \right)}\cdot N_{i}^{S}-\eta_{i}^{\mathrm{Sen}(\alpha )}\right|}{R_{i}^{S(\alpha )}}\right]}^{\frac{1}{m_{S}}}\mathrm{sgn}\left(-\xi_{i}^{\left(\alpha \right)}\cdot N_{i}^{S}-\eta_{i}^{\mathrm{Sen}(\alpha )}\right),$$

which is the governing equation for dislocation absorption by surfaces.

#### 2.4.3. GB Constitutive Relation: Slip Transmission

The key novelty of the present work is the consideration of slip transmission. To capture energetic and dissipative GB effects due to slip transmission, the GB microforce $\eta_{ABi}^{\mathrm{GB}(\alpha \beta )}$ is assumed to consist of an energetic part $\eta_{ABi}^{\mathrm{GBen}(\alpha \beta )}$ and a dissipative part $\eta_{ABi}^{\mathrm{GBdis}(\alpha \beta )}$

(39) $$\eta_{ABi}^{\mathrm{GB}(\alpha \beta )}=\eta_{ABi}^{\mathrm{GBen}(\alpha \beta )}+\eta_{ABi}^{\mathrm{GBdis}(\alpha \beta )}.$$

To consider the energetic GB effect, it is assumed that the power expended by $\eta_{ABi}^{\mathrm{GBen}(\alpha \beta )}$ at the GB equals the increase rate of GB energy ${\dot{\psi }}_{i}^{\mathrm{GB}}$ induced by the accumulation of GB defects resulting from slip transmission, namely

(40) $$\sum_{\alpha ,\beta }\int_{\Omega_{ABi}}\eta_{ABi}^{\mathrm{GBen}(\alpha \beta )}{\dot{\gamma }}_{AB}^{\left(\alpha \beta \right)}d\Omega_{ABi}-\int_{\Omega_{ABi}}{\dot{\psi }}_{i}^{\mathrm{GB}}d\Omega_{ABi}=0.$$

A simple quadratic form with respect to GB Burgers tensor $G_{AB}^{\left(\alpha \beta \right)}$ is adopted for the GB energy density such that ${\dot{\psi }}_{i}^{\mathrm{GB}}$ is expressed as

(41) $${\dot{\psi }}_{i}^{\mathrm{GB}}=\sum_{\alpha ,\beta ,\varphi ,\omega }\lambda_{AB}^{\mathrm{GB}(\alpha \beta \varphi \omega )}\left|M_{ABi}^{\left(\alpha \beta \right)}\right|\left|M_{ABi}^{\left(\varphi \omega \right)}\right|{\dot{\gamma }}_{AB}^{\left(\alpha \beta \right)}\gamma_{AB}^{\left(\varphi \omega \right)},$$

where $\left|t_{1}⊗t_{2}\right|=\sqrt{\left[t_{1}\cdot t_{1}\right]\left[t_{2}\cdot t_{2}\right]}$, and $\lambda_{AB}^{\mathrm{GB}(\alpha \beta \varphi \omega )}$ is the energetic GB hardening modulus. In Equation (41), the interaction between GB defects from different slip transmission processes are considered. Combing Equation (40) with Equation (41), one obtains the following constitutive relation for the energetic GB microforce $\eta_{ABi}^{\mathrm{GBen}(\alpha \beta )}$

(42) $$\eta_{ABi}^{\mathrm{GBen}(\alpha \beta )}=\sum_{\varphi ,\omega }\lambda_{AB}^{\mathrm{GB}(\alpha \beta \varphi \omega )}\left|M_{AB}^{\left(\alpha \beta \right)}\right|\left|M_{AB}^{\left(\varphi \omega \right)}\right|\gamma_{AB}^{\left(\varphi \omega \right)}.$$

The dissipative GB microforce $\eta_{ABi}^{\mathrm{GBdis}(\alpha \beta )}$ captures the resistance against slip transmission and is assumed to possess the following visco-plastic power-law relation

(43) $$\eta_{ABi}^{\mathrm{GBdis}(\alpha \beta )}=R_{AB}^{\mathrm{GB}(\alpha \beta )}{\left[\frac{{\dot{\gamma }}_{AB}^{\left(\alpha \beta \right)}}{{\dot{\gamma }}_{0}^{\mathrm{GB}}}\right]}^{m_{\mathrm{GB}}}\mathrm{sgn}\left({\dot{\gamma }}_{AB}^{\left(\alpha \beta \right)}\right),$$

where ${\dot{\gamma }}_{0}^{\mathrm{GB}}$ is the reference slip rate at the GB, and $m_{\mathrm{GB}}$ is the GB rate-sensitivity exponent. The GB resistance $R_{AB}^{\mathrm{GB}(\alpha \beta )}$ is expressed as

(44) $$R_{AB}^{\mathrm{GB}(\alpha \beta )}=R_{AB0}^{\mathrm{GB}(\alpha \beta )}+\sum_{\varphi ,\omega }H_{AB}^{\mathrm{GB}(\alpha \beta \varphi \omega )}{\left|G_{AB}^{\left(\varphi \omega \right)}\right|}_{\mathrm{acc}},$$

where ${\left|G_{AB}^{\left(\varphi \omega \right)}\right|}_{\mathrm{acc}}=\int \left|{\dot{G}}_{AB}^{\left(\varphi \omega \right)}\right|dt$ is the accumulated GB defect density, $R_{AB0}^{\mathrm{GB}(\alpha \beta )}$ is the initial GB resistance, and $H_{AB}^{\mathrm{GB}(\alpha \beta \varphi \omega )}$ is the dissipative GB hardening modulus. In Equation (44), the interaction between different slip transmission processes is considered.

By substituting Equation (43) into Equation (28), the microscopic GB force balance Equation (28) is rewritten as

(45) $$\begin{array}{cc} {\dot{\gamma }}_{AB}^{\left(\alpha \beta \right)}= & {\dot{\gamma }}_{0}^{\mathrm{GB}}{\left[\frac{\left|-\xi_{Ai}^{\left(\alpha \right)}\cdot N_{Ai}^{\mathrm{GB}}-D_{AB}^{\left(\alpha \beta \right)}\xi_{Bi}^{\left(\beta \right)}\cdot N_{Bi}^{\mathrm{GB}}-\eta_{ABi}^{\mathrm{GBen}(\alpha \beta )}\right|}{R_{AB}^{\mathrm{GB}(\alpha \alpha )}}\right]}^{\frac{1}{m_{\mathrm{GB}}}}\cdot \\ \; & \mathrm{sgn}\left(-\xi_{Ai}^{\left(\alpha \right)}\cdot N_{Ai}^{\mathrm{GB}}-D_{AB}^{\left(\alpha \beta \right)}\xi_{Bi}^{\left(\beta \right)}\cdot N_{Bi}^{\mathrm{GB}}-\eta_{ABi}^{\mathrm{GBen}(\alpha \beta )}\right), \end{array}$$

which is the governing equation for the slip transmission process between the incoming slip system α in grain *A* and the outgoing slip system β in grain *B* at the GB, where ${\dot{\gamma }}_{AB}^{\left(\alpha \beta \right)}$ is regarded as the measure of the rate of slip transmission.

The present GB model captures the influence of important factors including the misorientation between the incoming and outgoing slip systems, the GB orientation, GB defects and stress states at the GB which may affect slip transmission.

## 3. Numerical Example: A Bicrystal Thin Film Under Plane Constrained Shear

**Figure 2:**
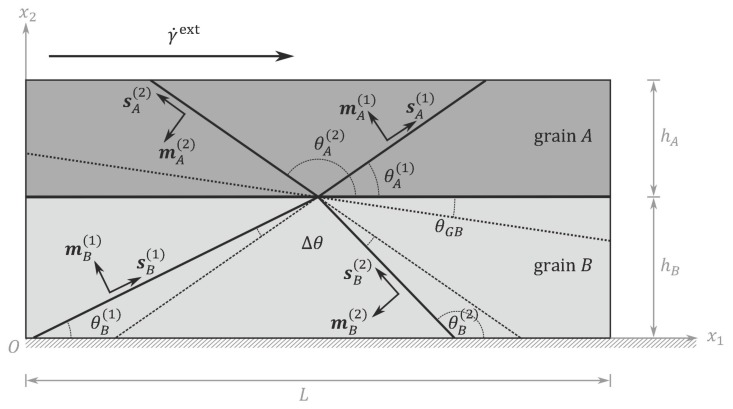
Sketch of the Benchmark Problem: A Bicrystal Thin Film under Plane Constrained Shear.

To illustrate influences of GB effects due to slip transmission on plastic behaviors in fine-grained polycrystals, the theory is implemented to study a plane strain benchmark problem of plane constrained shear of a bicrystal thin film. As depicted in Figure 2, the thin film consists of two grains *A* and *B* separated by a tilt GB parallel to the surfaces. Grain *I* (I=A,B) has a thickness $h_{I}$ in $x_{2}$-direction and is infinitely long in $x_{1}$-direction. For each grain, without loss of generality, two slip systems with each of them defined by a slip direction unit vector $s_{I}^{\left(\alpha \right)}=\left[\cos\theta_{I}^{\left(\alpha \right)},\sin\theta_{I}^{\left(\alpha \right)}\right]$ and a slip plane normal unit vector $m_{I}^{\left(\alpha \right)}=\left[-\sin\theta_{I}^{\left(\alpha \right)},\cos\theta_{I}^{\left(\alpha \right)}\right]$(α=1,2) are considered, where $\theta_{A}^{\left(2\right)}-\theta_{A}^{\left(1\right)}=\theta_{B}^{\left(2\right)}-\theta_{B}^{\left(1\right)}$. Following [38], the misorientation angle $\Delta \theta =\theta_{A}^{\left(\alpha \right)}-\theta_{B}^{\left(\alpha \right)}$ and, following [47], the rotation angle of the GB relative to the symmetry plane $\theta_{\mathrm{GB}}=\pi -\theta_{A}^{\left(1\right)}-\theta_{B}^{\left(2\right)}$ are introduced to measure the geometric mismatch between the grains. If $\theta_{\mathrm{GB}}=0$, the GB is symmetric. Crystallographic parameters including the grain misorientation, GB orientation, and grain size are varied to illustrate their influence on the plastic behavior of the thin film. Therefore, the considered bicrystal thin film is not specified to a certain material. The proposed model applies to general fine-grained polycrystalline metallic materials where the plasticity is mediated by dislocation activities.

The thin film suffers an externally prescribed shear rate ${\dot{\gamma }}^{\mathrm{ext}}$ with its lower surface being constrained such that the macroscopic boundary conditions are put down as

(46) $$\Delta u_{1}\left(x_{1},0\right)=\Delta u_{2}\left(x_{1},0\right)=\Delta u_{2}\left(x_{1},h\right)=0,\;\Delta u_{1}\left(x_{1},h\right)={\dot{\gamma }}^{\mathrm{ext}}h\Delta t,$$

where $\Delta u_{1}$ and $\Delta u_{2}$ denote the displacement increments after time increment Δt, and $h=h_{A}+h_{B}$. The continuity condition of the displacement at the GB reads

(47) $$u_{A1}\left(x_{1},h_{B}\right)=u_{B1}\left(x_{1},h_{B}\right),\;u_{A2}\left(x_{1},h_{B}\right)=u_{B2}\left(x_{1},h_{B}\right).$$

In the present plane strain problem, only edge GNDs are involved. Consider that slip transmission occurs at the GB. The corresponding relations between incoming and outgoing slip systems can be determined by taking advantage of slip transmission criteria [60,61], which is not elaborately pursued here. It is assumed that the incoming slip systems 1 and 2 in grain *A* correspond to the outgoing slip systems 1 and 2 in grain *B* respectively. Therefore, the microscopic boundary conditions at the GB are

(48) $${\dot{\gamma }}_{B}^{\left(1\right)}=D_{AB}^{\left(11\right)}{\dot{\gamma }}_{A}^{\left(1\right)}=D_{AB}^{\left(11\right)}{\dot{\gamma }}_{AB}^{\left(11\right)},$$

(49) $$\begin{array}{cc} {\dot{\gamma }}_{AB}^{\left(11\right)}= & {\dot{\gamma }}_{0}^{\mathrm{GB}}{\left[\frac{\left|-\xi_{Ai}^{\left(1\right)}\cdot N_{Ai}^{\mathrm{GB}}-D_{AB}^{\left(11\right)}\xi_{Bi}^{\left(1\right)}\cdot N_{Bi}^{\mathrm{GB}}-\eta_{ABi}^{\mathrm{GBen}(11)}\right|}{R_{AB}^{\mathrm{GB}(11)}}\right]}^{\frac{1}{m_{\mathrm{GB}}}}\cdot \\ \; & \mathrm{sgn}\left(-\xi_{Ai}^{\left(1\right)}\cdot N_{Ai}^{\mathrm{GB}}-D_{AB}^{\left(11\right)}\xi_{Bi}^{\left(1\right)}\cdot N_{Bi}^{\mathrm{GB}}-\eta_{ABi}^{\mathrm{GBen}(11)}\right), \end{array}$$

(50) $${\dot{\gamma }}_{B}^{\left(2\right)}=D_{AB}^{\left(22\right)}{\dot{\gamma }}_{A}^{\left(2\right)}=D_{AB}^{\left(22\right)}{\dot{\gamma }}_{AB}^{\left(22\right)},$$

(51) $$\begin{array}{cc} {\dot{\gamma }}_{AB}^{\left(22\right)}= & {\dot{\gamma }}_{0}^{\mathrm{GB}}{\left[\frac{\left|-\xi_{Ai}^{\left(2\right)}\cdot N_{Ai}^{\mathrm{GB}}-D_{AB}^{\left(22\right)}\xi_{Bi}^{\left(2\right)}\cdot N_{Bi}^{\mathrm{GB}}-\eta_{ABi}^{\mathrm{GBen}(22)}\right|}{R_{AB}^{\mathrm{GB}(22)}}\right]}^{\frac{1}{m_{\mathrm{GB}}}}\cdot \\ \; & \mathrm{sgn}\left(-\xi_{Ai}^{\left(2\right)}\cdot N_{Ai}^{\mathrm{GB}}-D_{AB}^{\left(22\right)}\xi_{Bi}^{\left(2\right)}\cdot N_{Bi}^{\mathrm{GB}}-\eta_{ABi}^{\mathrm{GBen}(22)}\right), \end{array}$$

where Equations (48) and (50) are the GB kinematic relations, and Equations (49) and (51) are the governing equations for slip transmission. In addition, in order to mimic the infinite extension of the thin film in $x_{1}$-direction, a representative part of the thin film with length *L* is considered, and periodic boundary conditions are imposed for displacements and GND densities, i.e.,

(52) $$\begin{array}{cccc} u_{1}\left(0,x_{2}\right) & =u_{1}\left(L,x_{2}\right), & u_{2}\left(0,x_{2}\right) & =u_{2}\left(L,x_{2}\right),\\ \rho^{\mathrm{ge}(1)}\left(0,x_{2}\right) & =\rho^{\mathrm{ge}(1)}\left(L,x_{2}\right), & \rho^{\mathrm{ge}(2)}\left(0,x_{2}\right) & =\rho^{\mathrm{ge}(2)}\left(L,x_{2}\right). \end{array}$$

The elastic parameters and the magnitude of the Burgers vector representative of aluminum are taken, i.e., μ=26.3GPa, ν=0.33 and $b^{\left(\alpha \right)}=0.286\;\mathrm{nm}$. Some of the plastic material parameters are chosen as $R_{0}^{b(\alpha )}=20\;\mathrm{MPa}$, $H^{b(\alpha \beta )}=200\;\mathrm{MPa}$, ${\dot{\gamma }}_{0}^{b}={\dot{\gamma }}_{0}^{\mathrm{GB}}=0.001\;s^{-1}$, and $m_{b}=m_{\mathrm{GB}}=0.05$. The initial GND densities are assumed to be zero. The characteristic length scale is assumed to be constant, i.e., l=0.5μm. The magnitude of the loading shear rate ${\dot{\gamma }}^{\mathrm{ext}}$ is $0.001\;s^{-1}$. Since attention is restricted to the new GB model, unless otherwise mentioned, the surface is considered to be impenetrable such that plastic slips vanish at the surface. For simplicity, the interaction between the two slip transmission processes is ignored by taking $\lambda_{AB}^{\mathrm{GB}(1122)}=\lambda_{AB}^{\mathrm{GB}(2211)}=0$ and $H_{AB}^{\mathrm{GB}(1122)}=H_{AB}^{\mathrm{GB}(2211)}=0$. To simplify the discussion, GB parameters for the two slip transmission process are assumed to be the same, i.e., $\lambda_{AB}^{\mathrm{GB}(1111)}=\lambda_{AB}^{\mathrm{GB}(2222)}=\lambda^{\mathrm{GB}}$, $R_{AB0}^{\mathrm{GB}(11)}=R_{AB0}^{\mathrm{GB}(22)}=R_{0}^{\mathrm{GB}}$, $H_{AB}^{\mathrm{GB}(1111)}=H_{AB}^{\mathrm{GB}(2222)}=H^{\mathrm{GB}}$. In addition, the average distance between slip planes of the incoming slip system is assumed to equal that of the corresponding outgoing slip system such that $D_{AB}^{\left(11\right)}=D_{AB}^{\left(22\right)}=1$. The simulations are done by using an in-house finite element code, see Appendix A for details about the finite element implementation.

### 3.1. Influence of Energetic and Dissipative GB Parameters

**Figure 3:**
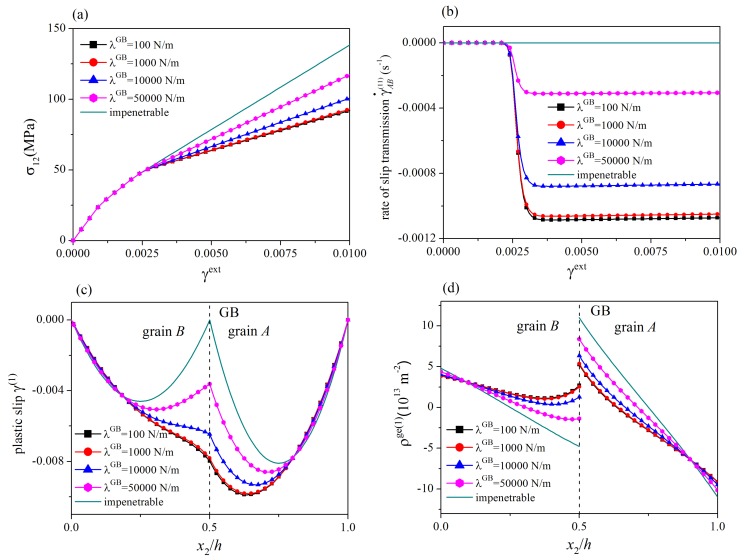
Influence of energetic GB hardening modulus: (**a**) stress-strain curves; (**b**) the rate of slip transmission; (**c**) distribution of plastic slip at $\gamma^{\mathrm{ext}}=0.01$; (**d**) distribution of GND density at $\gamma^{\mathrm{ext}}=0.01$. The critical load for the onset of slip transmission depends on the initial GB resistance $R_{0}^{\mathrm{GB}}$. For decreasing energetic GB hardening modulus $\lambda^{\mathrm{GB}}$, the steady-state value of the rate of slip transmission increases, the strain hardening rate after the onset of slip transmission decreases, and the curve of distribution of plastic slip becomes smoother at the GB.

**Figure 4:**
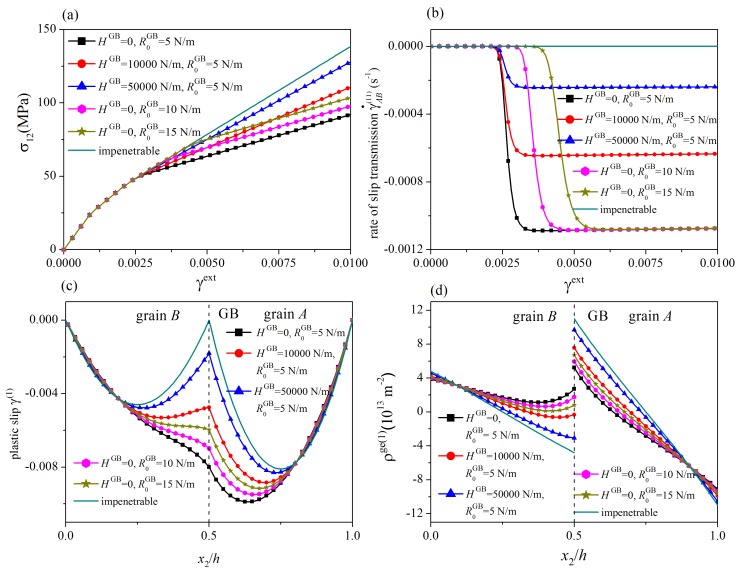
Influence of dissipative GB parameters: (**a**) stress-strain curves; (**b**) the rate of slip transmission; (**c**) distribution of plastic slip at $\gamma^{\mathrm{ext}}=0.01$; (**d**) distribution of GND density at $\gamma^{\mathrm{ext}}=0.01$. For decreasing dissipative GB hardening modulus $H^{\mathrm{GB}}$, the steady-state value of the rate of slip transmission increases, the strain hardening rate after the onset of slip transmission decreases, and the curve of distribution of plastic slip becomes smoother at the GB.

In this section, the influences of the energetic and dissipative GB parameters are addressed. A symmetric tilt GB with $\theta_{A}^{\left(1\right)}=\theta_{A}^{\left(2\right)}-60{}^{\circ}=70{}^{\circ}$, $\theta_{B}^{\left(1\right)}=\theta_{B}^{\left(2\right)}-60{}^{\circ}=50{}^{\circ}$, Δθ=20°, and $\theta_{\mathrm{GB}}=0{}^{\circ}$ is considered. The values of grain thickness are taken as $h_{A}=h_{B}=1\;\mu m$. For different values of the energetic GB hardening modulus $\lambda^{\mathrm{GB}}$, stress-strain curves, the evolution of the rate of slip transmission, the distribution of plastic slip at $\gamma^{\mathrm{ext}}=0.01$, and the distribution of GND density at $\gamma^{\mathrm{ext}}=0.01$ are plotted in Figure 3, where $R_{0}^{\mathrm{GB}}=5\;N/m$ and $H^{\mathrm{GB}}=0$. Since the results of slip systems 1 and 2 are similar, only those of the slip system 1 are shown. In addition, the results for impenetrable GBs at which plastic slips vanish are also given for comparison. The corresponding results for different values of the dissipative GB hardening modulus and the initial GB slip resistance are displayed in Figure 4, where $\lambda^{\mathrm{GB}}=0$.

In Figure 3a and Figure 4a, on each stress-strain curve (curves with symbols), two yielding points are observed. The first one is the traditional bulk yielding point. The second one denotes the onset of slip transmission after which the rate of slip transmission immediately increases from zero to a steady-state value (see Figure 3b and Figure 4b). From Figure 4a,b, the critical load for the onset of slip transmission is governed by the initial GB resistance $R_{0}^{\mathrm{GB}}$ in a way that a larger $R_{0}^{\mathrm{GB}}$ gives rise to a larger critical load. As slip transmission weakens the dislocation pile-up at the GB, the strain hardening rate decreases after the onset of slip transmission. With the increase of the energetic and dissipative GB hardening modulus $\lambda^{\mathrm{GB}}$ and $H^{\mathrm{GB}}$, it is more and more difficult for dislocations to penetrate the GB. Consequently, the steady-state value of the rate of slip transmission decreases, and the strain hardening rate at the stage with slip transmission increases. As shown in Figure 3c,d and Figure 4c,d, for a smaller GB hardening modulus and/or a smaller initial GB resistance, the curve of distribution of plastic slip is smoother at the GB, and the GND density at the GB is smaller, indicating that slip transmission occurs more easily. In addition, if the initial GB slip resistance and/or the GB hardening modulus approach infinity, the present GB model reduces to the impenetrable GB model. The tendency of the stress-strain curves and the dependence of the strain hardening rate on the GB hardening modulus are consistent with those predicted by existing models [37,38,39,47,49].

### 3.2. Influence of Grain Size

**Figure 5:**
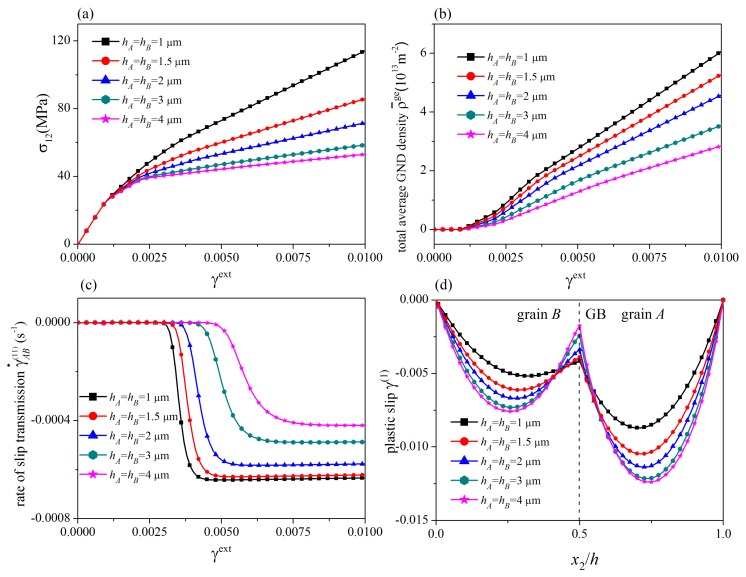
Size effects: (**a**) stress-strain curves; (**b**) total average GND density; (**c**) the rate of slip transmission; (**d**) distribution of plastic slip at $\gamma^{\mathrm{ext}}=0.01$. The flow stress and the total average GND density at a specified load increase with the decrease of grain size. For a film with a smaller grain size, the critical load of onset of slip transmission is smaller, and the steady-state value of slip transmission rate is larger, indicating that the occurrence of slip transmission is easier.

To investigate the influence of grain size, for different values of grain thickness, the stress-strain curve, the evolution of the total average GND density ${\bar{\rho }}^{\mathrm{ge}}=\int_{0}^{h}\left[\left|\rho^{\mathrm{ge}(1)}\right|+\left|\rho^{\mathrm{ge}(2)}\right|\right]dx_{2}/h$, the evolution of the rate of slip transmission, and the distribution of plastic slip at $\gamma^{\mathrm{ext}}=0.01$ are plotted in Figure 5a–d, where GB parameters are taken as $\lambda^{\mathrm{GB}}=100\;N/m$, $R_{0}^{\mathrm{GB}}=10\;N/m$, and $H^{\mathrm{GB}}=10,000\;N/m$, and the orientation angles of slip systems are $\theta_{A}^{\left(1\right)}=\theta_{A}^{\left(2\right)}-60{}^{\circ}=70{}^{\circ}$ and $\theta_{B}^{\left(1\right)}=\theta_{B}^{\left(2\right)}-60{}^{\circ}=50{}^{\circ}$ with Δθ=20° and $\theta_{\mathrm{GB}}=0{}^{\circ}$. By comparing the results of thin films with different values of grain thickness, it is seen that the plastic behavior is significantly size-dependent. From Figure 5a,b, for a specified load $\gamma^{\mathrm{ext}}$, a smaller grain thickness results in a larger flow stress and a larger total average GND density. The size-dependence of the total average GND density in Figure 5b is qualitatively consistent with the corresponding result by the DDD simulation in [20]. As displayed in Figure 5a,c, for the thin film with a smaller grain thickness, the critical load for the onset of slip transmission is smaller, and the steady-state value of the rate of slip transmission is larger, implying that the occurrence of slip transmission is easier. From Figure 5d, with the decrease of grain thickness, the plastic slip decreases in the middle of grains but increases near the GB. This is due to the fact that in the middle, the plastic slip is governed by dislocation interaction which is stronger if the grain thickness is smaller, while, near the GB, the plastic slip is dominated by slip transmission which is easier to occur in the film with a smaller grain thickness.

### 3.3. Influence of Grain Misorientation and GB Orientation

**Figure 6:**
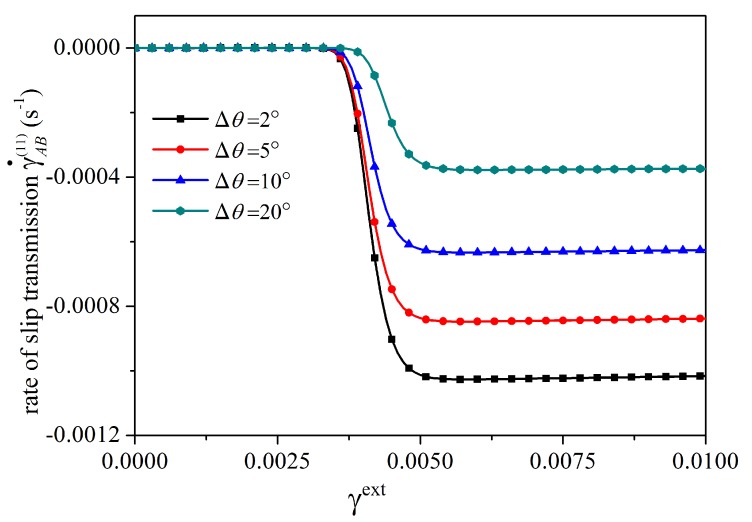
Influence of grain misorientation on the rate of slip transmission. For a smaller grain misorientation angle, the steady-state value of slip transmission is larger, implying that the occurrence of slip transmission is easier.

Firstly, the influence of grain misorientation is examined. To this end, four different values of Δθ, i.e., 2°, 5°, 10°, and 20° are considered. The corresponding orientation angles of the slip systems are $\theta_{A}^{\left(1\right)}=60{}^{\circ}+\Delta \theta /2$, $\theta_{B}^{\left(1\right)}=60{}^{\circ}-\Delta \theta /2$, $\theta_{A}^{\left(1\right)}=120{}^{\circ}+\Delta \theta /2$, and $\theta_{B}^{\left(2\right)}=120{}^{\circ}-\Delta \theta /2$ with $\theta_{\mathrm{GB}}=0{}^{\circ}$. The GB parameters are taken as $\lambda^{\mathrm{GB}}=20,000\;N/m$, $R_{0}^{\mathrm{GB}}=15\;N/m$, and $H^{\mathrm{GB}}=10,000\;N/m$. In Figure 6, for different Δθ, the evolution of the rate of slip transmission is plotted. It is seen that the steady-state value of the rate of slip transmission increases with the decrease of grain misorientation angle Δθ. This indicates that slip transmission is more likely to occur if the grain misorientation angle is smaller, which is in accordance with experimental observations [62].

**Figure 7:**
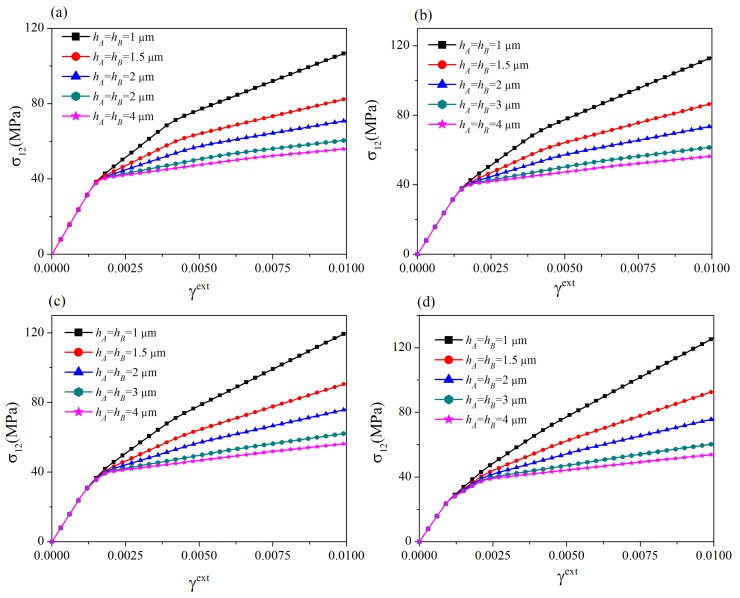
Size-dependent stress-strain curves: (**a**) Δθ=2°; (**b**) Δθ=5°; (**c**) Δθ=10°; (**d**) Δθ=20°. For thin films with smaller grain misorientation angles, the strain hardening rate significantly decreases after the onset of slip transmission.

Figure 7 plots the grain size-dependence of the stress-strain curves for those four grain misorientation angles. Comparing the different cases, one can conclude that the influence of grain misorientation on the size-dependence of strain hardening rate after the onset of slip transmission is vital. In the case with a smaller grain misorientation angle (Δθ=2° or Δθ=5°), as slip transmission easily occurs, the dislocation pile-up at the GB is significantly relieved. Consequently, the strain hardening rate obviously decreases compared to the previous stage without slip transmission. Conversely, in the case with a larger grain misorientation angle (Δθ=10° or Δθ=20°), as slip transmission is difficult to proceed due to the strong GB resistance, the change of the strain hardening rate is trivial. In addition, for a given grain size, the larger the grain misorientation, the larger the flow stress at a specified load. These results are qualitatively consistent with the corresponding DDD simulation results in [18].

**Figure 8:**
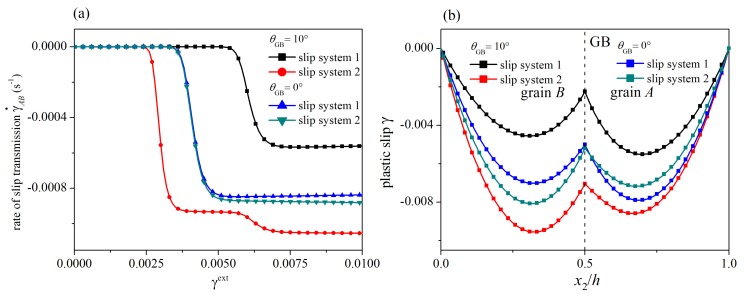
Influence of grain boundary orientation: (**a**) the rate of slip transmission; (**b**) distribution of plastic slip at $\gamma^{\mathrm{ext}}=0.01$. The GB orientation significantly affects the slip transmission behavior.

Then, the influence of the GB orientation is illustrated. To this end, a symmetric GB ($\theta_{\mathrm{GB}}=0{}^{\circ}$) and an asymmetric GB ($\theta_{\mathrm{GB}}=10{}^{\circ}$) are considered. The corresponding orientation angles of the slip systems are $\theta_{A}^{\left(1\right)}=62.5{}^{\circ}-\theta_{\mathrm{GB}}$, $\theta_{B}^{\left(1\right)}=57.5{}^{\circ}-\theta_{\mathrm{GB}}$, $\theta_{A}^{\left(2\right)}=122.5{}^{\circ}-\theta_{\mathrm{GB}}$, and $\theta_{B}^{\left(2\right)}=117.5{}^{\circ}-\theta_{\mathrm{GB}}$ with Δθ=5°. In Figure 8a,b, the evolution of the rate of slip transmission and the distribution of plastic slip at $\gamma^{\mathrm{ext}}=0.01$ for the two cases are plotted. The GB orientation significantly affects slip transmission. For the case with a symmetric GB ($\theta_{\mathrm{GB}}=0{}^{\circ}$), the critical loads for the onset of the two slip transmission processes are almost the same, and the difference in the steady-state value of the rate of slip transmission is small. For the case with an asymmetric GB ($\theta_{\mathrm{GB}}=10{}^{\circ}$), the distribution of plastic slip and the evolution of the rate of slip transmission for the slip transmission process 1 obviously differ from those for the slip transmission process 2. From Figure 8b, the slip transmission process 2 occurs first. After the onset of the slip transmission process 1, the rate of slip transmission for the slip transmission process 2 suffers a sudden increase.

### 3.4. Influence of Surface Constraint

**Figure 9:**
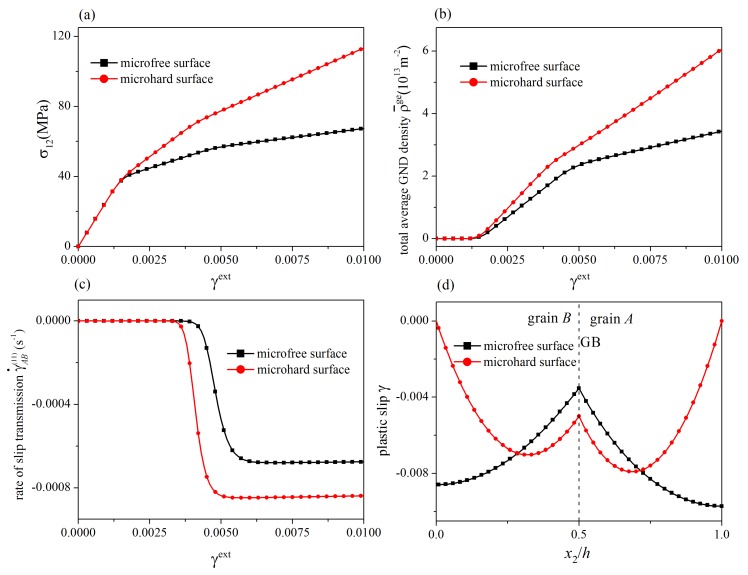
Influence of surface constraint: (**a**) stress-strain curves; (**b**) total average GND density; (**c**) the rate of slip transmission; (**d**) distribution of plastic slip at $\gamma^{\mathrm{ext}}=0.01$. Dislocation absorption by surfaces and slip transmission may compete with each other.

In the above discussion, in order to illustrate the GB influence separately, the microhard (impenetrable) surface which is completely constrained is considered. However, if the surface is permitted to absorb dislocations, slip transmission processes at the GB are also affected. Thus, the influence of surface constraint is investigated here. A microhard surface and a microfree surface freely penetrable by dislocations representing two extreme cases are considered. It is pointed out that the actual surface being in-between should be modeled by the surface model in Section 2. The orientation angles of the slip systems are $\theta_{A}^{\left(1\right)}=62.5{}^{\circ}$, $\theta_{B}^{\left(1\right)}=57.5{}^{\circ}$, $\theta_{A}^{\left(2\right)}=122.5{}^{\circ}$, and $\theta_{B}^{\left(2\right)}=117.5{}^{\circ}$ with $\theta_{\mathrm{GB}}=0{}^{\circ}$ and Δθ=5°. The GB parameters are $\lambda^{\mathrm{GB}}=20,000\;N/m$, $R_{0}^{\mathrm{GB}}=15\;N/m$, and $H^{\mathrm{GB}}=10,000\;N/m$. Stress-strain curves, the evolution of the total average GND density, the evolution of the rate of slip transmission, and the distribution of plastic slip at $\gamma^{\mathrm{ext}}=0.01$ for the two cases are shown in Figure 9. From Figure 9a,c, for the case with a microhard surface, the critical load for the onset of slip transmission is smaller and the steady-state value of the rate of slip transmission is larger, indicating the slip transmission is easier to occur. It is attributed to the fact that in the case with a microhard surface, since dislocations are not absorbed by surfaces, dislocation pile-up at the GB is stronger, giving rise to a larger driving force for slip transmission. Due to the surface constraint, in the case with a microhard surface, the decrease of the strain hardening rate and the increase rate of average GND densities after slip transmission is trivial compared to the case with a microfree surface. From Figure 9d, as the governing mechanism for the development of plastic slip near the surface and that near the GB are dislocation absorption by surfaces and slip transmission respectively, in the case with a microhard surface, plastic slip near the surface is smaller but that near the GB is larger. It reflects the competition between dislocation absorption by surfaces and slip transmission.

## 4. Summary

In this work, a GB model with consideration of slip transmission is proposed within the framework of finite deformation gradient polycrystal plasticity. In the GB model, for each slip transmission process, there are a GB kinematic relation between the plastic slip of the incoming slip system and that of the outgoing slip system at the GB and a governing equation for slip transmission, which constitute the GB microscopic boundary conditions. Both the energetic and the dissipative GB effects are considered by introducing an energetic and a dissipative GB microforce for each slip transmission process. By properly constructing energetic and dissipative GB constitutive relations, the important factors including grain misorientation, the GB orientation, GB defects and stress state at the GB which may affect slip transmission are captured in the present GB model. The main advantage of the present model over the existing models is the consideration of underlying physical mechanisms of slip transmission.

The mathematical framework of the present rate-dependent model is a fully coupled, strongly nonlinear initial boundary value problem with non-standard boundary conditions, the numerical implementation of which is done via a dual-mixed nonlinear finite element method. The treatment of microscopic GB conditions is emphasized. A benchmark problem of a bicrystal thin film under plane constrained shear is studied. It is found that the GB parameter, grain size, grain misorientation, GB orientation, and surface constraint can significantly affect the slip transmission and the plastic behavior of thin films. Particularly, in thin films with a smaller grain size and/or a smaller grain misorientation, slip transmission occurs more easily. These results predicted by the present model qualitatively agree with some experimental observations and results of DDD simulations in the literature.

## Appendix A

To realize the numerical implementation of the model, a dual-mixed finite element formulation put forward by [63] is adopted. The balance of momentum (24) and the evolution Equations (8) and (9) of the GND densities are treated as governing equations such that the displacement u and the GND densities $\rho_{i}^{\mathrm{ge}(\alpha )}$ and $\rho_{i}^{\mathrm{gs}(\alpha )}$ act as global variables. The plastic slip $\gamma^{\left(\alpha \right)}$ is evaluated locally at the integration points by the evolution equation of the plastic slip in the bulk and that at the GB/surface. The treatment for the balance of momentum is standard. Thus, attention is restricted to the discretization and implementation of the evolution equations of the GND densities. As the evolution equations of the edge GND density and the screw GND density are similar, only the former is explained for the sake of brevity. To obtain its weak forms, the governing equation of the edge GND density is multiplied by the weight function $\delta \rho_{i}^{\mathrm{ge}(\alpha )}$ and then integrated over the volume, followed by the application of divergence theorem, which yields

(A1) $$\begin{array}{cc} \; & \int_{V_{i}}\left[\delta {\dot{\rho }}_{i}^{\mathrm{ge}(\alpha )}{\dot{\rho }}_{i}^{\mathrm{ge}(\alpha )}-\frac{1}{b^{\left(\alpha \right)}}s^{\left(\alpha \right)}\cdot \nabla_{i}\delta {\dot{\rho }}_{i}^{\mathrm{ge}(\alpha )}{\dot{\gamma }}^{\left(\alpha \right)}\right]dV_{i}\\ \; & +\int_{S_{i}}\frac{1}{b^{\left(\alpha \right)}}s^{\left(\alpha \right)}\cdot N_{i}^{S}\delta {\dot{\rho }}_{i}^{\mathrm{ge}(\alpha )}{\dot{\gamma }}^{\left(\alpha \right)}dS_{i}\\ \; & +\int_{\Omega_{ABi}}\frac{1}{b_{A}^{\left(\alpha \right)}}s_{A}^{\left(\alpha \right)}\cdot N_{Ai}^{\mathrm{GB}}\delta {\dot{\rho }}_{Ai}^{\mathrm{ge}(\alpha )}\sum_{\beta }{\dot{\gamma }}_{AB}^{\left(\alpha \beta \right)}d\Omega_{ABi}\\ \; & +\int_{\Omega_{ABi}}\frac{1}{b_{B}^{\left(\alpha \right)}}s_{B}^{\left(\alpha \right)}\cdot N_{Bi}^{\mathrm{GB}}\delta {\dot{\rho }}_{Bi}^{\mathrm{ge}(\alpha )}\sum_{\beta }D_{AB}^{\left(\beta \alpha \right)}{\dot{\gamma }}_{AB}^{\left(\beta \alpha \right)}d\Omega_{ABi}=0, \end{array}$$

where the GB kinematic relation (14) is considered. For the spatial discretization, the volume of the body is divided into finite elements, in each of which, following the standard Galerkin approach, the unknown fields of the displacement and GND densities and the weight functions are approximated by their nodal values multiplied by the shape functions. In addition, the adjacent elements on the two sides of the GB are decoupled by placing double nodes at a GB nodal point. To satisfy the continuity condition of the displacement at the GB, the displacement of those node pairs are forced to be equal. For the benchmark problem in the present work, 4-nodes bilinear quadrilateral elements with 2×2 full integration are chosen for the spatial discretization of both the displacement and the GND densities. To evaluate the GB/surface integration, two additional integration points are placed on each surface/GB edge of elements at the GB/surface.

For the time integration within a time increment $\left[t_{n},t_{n+1}\right]$, the implicit backward Euler scheme is adopted such that

(A2) $$\begin{array}{c} {\dot{\gamma }}_{n+1}^{\left(\alpha \right)}=\frac{\Delta \gamma^{\left(\alpha \right)}}{\Delta t},\;{\dot{\rho }}_{n+1}^{\mathrm{ge}(\alpha )}=\frac{\Delta \rho^{\mathrm{ge}(\alpha )}}{\Delta t},\\ F_{n+1}^{p-1}=F_{n}^{p-1}\cdot \left[I-\sum_{\alpha }\Delta \gamma^{\left(\alpha \right)}s^{\left(\alpha \right)}⊗m^{\left(\alpha \right)}\right], \end{array}$$

where $\Delta \gamma^{\left(\alpha \right)}=\gamma_{n+1}^{\left(\alpha \right)}-\gamma_{n}^{\left(\alpha \right)}$, $\Delta \rho^{\mathrm{ge}(\alpha )}=\rho_{n+1}^{\mathrm{ge}(\alpha )}-\rho_{n}^{\mathrm{ge}(\alpha )}$, $\Delta t=t_{n+1}-t_{n}$ and the subscript *n* identifies the quantities at the nth time step.

For the incorporation of the GB/surface contributions, the evaluation of the increment of the plastic slip at the GB/surface integration points is required. As discussed in [52], at the surface, the increment of the plastic slip $\Delta \gamma^{\left(\alpha \right)}$ is determined by the governing Equation (38) for dislocation absorption by surfaces

(A3) $$\Delta \gamma^{\left(\alpha \right)}=\Delta t{\dot{\gamma }}_{0}^{S}{\left[\frac{\left|-\xi_{i}^{\left(\alpha \right)}\cdot N_{i}^{S}-\eta_{i}^{\mathrm{Sen}(\alpha )}\right|}{R_{i}^{S(\alpha )}}\right]}^{\frac{1}{m_{s}}}\mathrm{sgn}\left(-\xi_{i}^{\left(\alpha \right)}\cdot N_{i}^{S}-\eta_{i}^{\mathrm{Sen}(\alpha )}\right).$$

The plastic slips required to evaluate the terms on the right hand side are extrapolated from the bulk. Accordingly, $\Delta \gamma_{AB}^{\left(\alpha \beta \right)}$ at the GB integration points is evaluated by the governing Equation (45) for slip transmission

(A4) $$\begin{array}{cc} \Delta \gamma_{AB}^{\left(\alpha \beta \right)}= & \Delta t{\dot{\gamma }}_{0}^{\mathrm{GB}}{\left[\frac{\left|-\xi_{Ai}^{\left(\alpha \right)}\cdot N_{Ai}^{\mathrm{GB}}-D_{AB}^{\left(\alpha \beta \right)}\xi_{Bi}^{\left(\beta \right)}\cdot N_{Bi}^{\mathrm{GB}}-\eta_{ABi}^{\mathrm{GBen}(\alpha \beta )}\right|}{R_{AB}^{\mathrm{GB}(\alpha \beta )}}\right]}^{\frac{1}{m_{\mathrm{GB}}}}\cdot \\ \; & \mathrm{sgn}\left(-\xi_{Ai}^{\left(\alpha \right)}\cdot N_{Ai}^{\mathrm{GB}}-D_{AB}^{\left(\alpha \beta \right)}\xi_{Bi}^{\left(\beta \right)}\cdot N_{Bi}^{\mathrm{GB}}-\eta_{ABi}^{\mathrm{GBen}(\alpha \beta )}\right), \end{array}$$

where the plastic slips required to calculate the quantities on the right hand side are extrapolated from the interior of grain *A* or grain *B*. Since quantities from both grains are involved in Equation (A4), the adjacent elements on the two sides of the GB are coupled, by which intergranular interactions between dislocations are considered.

By combing the spatial and temporal discretization, the weak forms of the governing equations reduce to a highly nonlinear, strongly coupled system of equations at each time step, which is solved numerically by a Newton-Raphson solution strategy.
